# MR-guided radiotherapy for prostate cancer: state of the art and future perspectives

**DOI:** 10.1259/bjr.20210800

**Published:** 2022-03-09

**Authors:** Kobika Sritharan, Alison Tree

**Affiliations:** 1 The Royal Marsden NHS Foundation Trust, London, United Kingdom; 2 The Institute of Cancer Research, London, United Kingdom

## Abstract

Advances in radiotherapy technology have increased precision of treatment delivery and in some tumour types, improved cure rates and decreased side effects. A new generation of radiotherapy machines, hybrids of an MRI scanner and a linear accelerator, has the potential to further transform the practice of radiation therapy in some cancers. Facilitating superior image quality and the ability to change the dose distribution online on a daily basis (termed “daily adaptive replanning”), MRI-guided radiotherapy machines allow for new possibilities including increasing dose, for hard to treat cancers, and more selective sparing of healthy tissues, where toxicity reduction is the key priority.

These machines have already been used to treat most types of cancer, although experience is still in its infancy. This review summarises the potential and current evidence for MRI-guided radiotherapy, with a predominant focus on prostate cancer. Current advantages and disadvantages are discussed including a realistic appraisal of the likely potential to improve patient outcomes. In addition, horizon scanning for near-term possibilities for research and development will hopefully delineate the potential role for this technology over the next decade.

## MR-guided radiotherapy

In recent decades, advances in imaging and technology have led to improvements in target coverage and conformality in addition to normal tissue sparing in radiotherapy treatment delivery. From 3D conformal radiotherapy to intensity-modulated radiotherapy (IMRT), volumetric arc therapy (VMAT) and stereotactic body radiotherapy (SBRT), radiotherapy planning and delivery has become increasingly complex.

The benefits of integrating MRI into the radiotherapy treatment pathway has been reported as early as 1986.^1^ These include, but are not limited to, superior soft tissue contrast, the lack of ionising radiation, the ability to acquire non-invasive functional imaging and the possibility of real-time imaging during beam delivery. Most experience of MR in radiotherapy so far has been with respect to improved target delineation on MRI.

Image-guided radiotherapy (IGRT) has been a key development in the delivery of radiotherapy and is now utilised in most radiotherapy treatments. It refers to imaging that is taken in the treatment room at the start of, or during, each fraction followed by individual positional adjustments to increase accuracy and ensure the planned dose is delivered to the target.^2^ The most recent technological advancement is the creation of MRI-radiotherapy hybrid systems, by virtue of its superior image quality. MRI-guided RT (MRIgRT) allows the possibility of acquiring MR images at any time point during the radiotherapy treatment and its implementation and use is rapidly expanding.

Radiotherapy is used in the treatment of half of all patients with cancer and cures up to 40% of patients.^3^ Prostate cancer is the most common cancer in the UK, with over 47,000 men diagnosed each year, accounting for over a quarter of all new male cancer diagnoses.^4^ The majority of patients diagnosed with localised disease are treated with external beam radiotherapy (EBRT). As the α/β ratio of prostate is low, at <2 Gy,^5^ many trials have proven hypofractionation (around 3 Gy per fraction) to be non-inferior to standard fractionation (2 Gy per fraction).^6,7^ There is now a body of Level II evidence suggesting that ultrahypofractionation with 5 fractions may be equivalent to 20 fraction treatments. This is being tested in the PACE B^8^ and PACE C trials.

This review explores the current role of the MR-Linac in clinical practice, its benefits, limitations, and potential role in the future, with a focus on prostate cancer.

### MRIgRT systems

Multiple MRIgRT systems are available with varying magnetic field strengths (Table 1), of which only two are being used in a clinical setting.^9,10^ Strengths and limitations of MR linac systems are described in Table 2.

In 2014, the Viewray MRIdian system (ViewRay Inc, Oakwood Village, OH) was the first to be used to treat patients, combining a tricobalt-60 source with a 0.35T MR imaging system and since 2017, a 6-Megavoltage Linac with a 0.35T MRI.^11,12^ The Elekta Unity system (AB, Stockholm, Sweden) has been in clinical use since 2017 and has a 1.5T imaging system which is integrated with a 7-Megavoltage linear accelerator.^13,14^ Two other systems currently in development are being used primarily for research; an Australian^15^ and a Canadian system.^16^

**Table 1.** MRIgRT systems currently available, either commercially or for research purposes

MR-RT system	Imaging strength	Linac	Bore size
Viewray MRIdian system (12) (Viewray Technologies Inc, Oakwood Village, OH)	0.35T	Integrates either tricobalt-60 or 6 MV linac	70 cm closed bore
Elekta MR-Linac (13) (Elekta AB, Stockholm, Sweden)	1.5T	7 MV	70 cm closed bore
Sydney Inline Australian system (15) (Australian MRI-Linac Program)	1.0T	6 MV	82 cm open bore
Aurora RT system (16) (MagnetTx, Alberta, Canada)	0.6T	6 MV	60 cm

MRIgRT, MRI-guided RT.

**Table 2.** This table summarises the advantages and limitations of the MR-Linac. Each point is described in more detail in the text.

Benefits	Limitations
Superior image quality compared to CT	Technical:- Geometric distortions and artefacts can impact the MR image quality. Electron return effect. Lack of non-coplanar & electron beams
Online adaptive radiotherapy	Limitations with bore size & craniocaudal field size length
Real-time cross-sectional imaging	Longer treatment times. Noisy during imaging. Not suitable for claustrophobic patients
No additional radiation exposure with imaging	Multi-disciplinary team needed to deliver treatment daily
Additional MR imaging possible daily during treatment including functional imaging	E Currently a research tool

MDT, multidisciplinary team.

## State of the art technology: advantages and limitations

### Advantages

#### Superior image quality

The PTV (planning target volume) encompasses the target requiring treatment plus a margin to account for setup and patient movement error. This margin can be up to 15 mm for some radical treatments and will inevitably encompass surrounding healthy tissue. Target delineation is said to be the weakest link in the delivery of accurate radiotherapy.^17^

**Figure 1.**
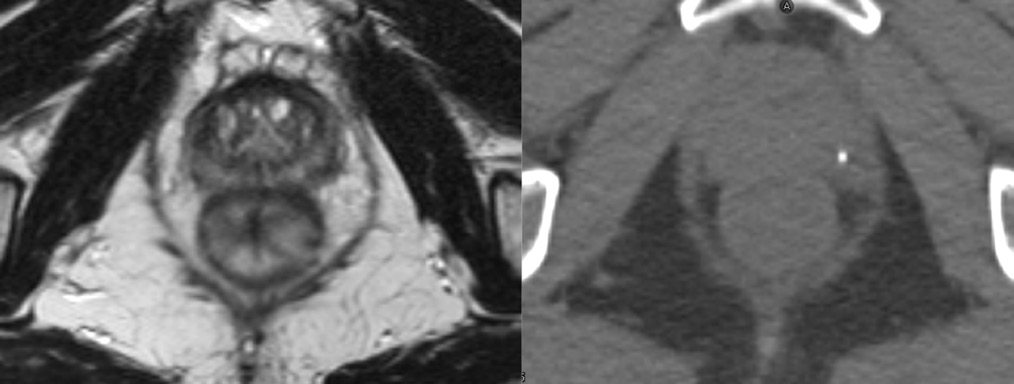
Axial images of an MRI of the prostate (left) and CT of the prostate (right) . The architecture and boundaries of the prostate are more clearly visualised on the MRI due to superior soft tissue contrast.

The primary advantage of an MR-integrated radiotherapy system is that of superior soft tissue contrast when compared to X-ray-based imaging.^18^ This is highlighted in Figure 1, which displays the difference between MR and CT when visualising soft tissue anatomy; the prostate and surrounding tissues, and their interfaces, are more clearly demarcated on the MRI. In prostate cancer, the clinical target volume drawn on MR images has been shown to be smaller, by around 30%,^19^ and more consistent when compared to CT-derived contours.^20,21^ Contouring on MRIs has been shown to reduce interobserver variability in prostate cancer^22^ and improve precision in other tumour sites such as brain, nasopharynx as well as with critical structures such as the brachial plexus.^23^ Therefore, with a clearer anatomical picture the volume of normal tissue irradiated could be reduced, due to a combination of smaller volumes and margins.^24^ The smaller volumes could lead to a reduction in treatment toxicity.^25,26^

In addition, precise delivery of radiation dose to the PTV during the course of treatment is dependent on visualisation of the organs. Current IGRT techniques include kV-imaging and CBCTs, which can be affected by motion artefacts and poor tissue contrast.^9,20^

Whether the superior image quality of MRgRT truly offers a therapeutic benefit, when compared to the use of current image guidance techniques such as fiducials and cone beam CTs used with standard linacs, is yet to be seen. This is particularly pertinent in prostate cancer, where rates of cure are high and that of toxicity is low even with standard techniques.

#### Online adaptive radiotherapy

One of the unique features of MRIgRT systems is the integration of online adaptive radiotherapy (ART). Although current IGRT techniques allow couch corrections to account for interfractional changes, it does not account for complex geometric changes such as target rotation, deformation and weight loss.^27^ Due to poorer soft tissue image quality, the accuracy of current IGRT techniques is limited and often large PTV margins are applied to account for this.

In prostate cancer, movement of the seminal vesicles and lymph nodes may occur independent to the prostate and, if in conflict, the prostate is prioritised, potentially missing the other targets.^28^ Interestingly, a study demonstrated that approximately a third of fractions would benefit from replanning, when the original plan is overlayed on the daily CBCT due to the difference in delivered dose compared to the planned dose.^9,29^ In some tumour types such as the brain, there is no evidence that conventional IGRT is inferior to MRIgRT as intrafraction motion is negligible and thus, using X-ray based localisation is likely to be sufficient. MRIgRT provides new possibilities of biologically targeted adaptive dose delivery and these are currently being tested.

**Figure 2.**
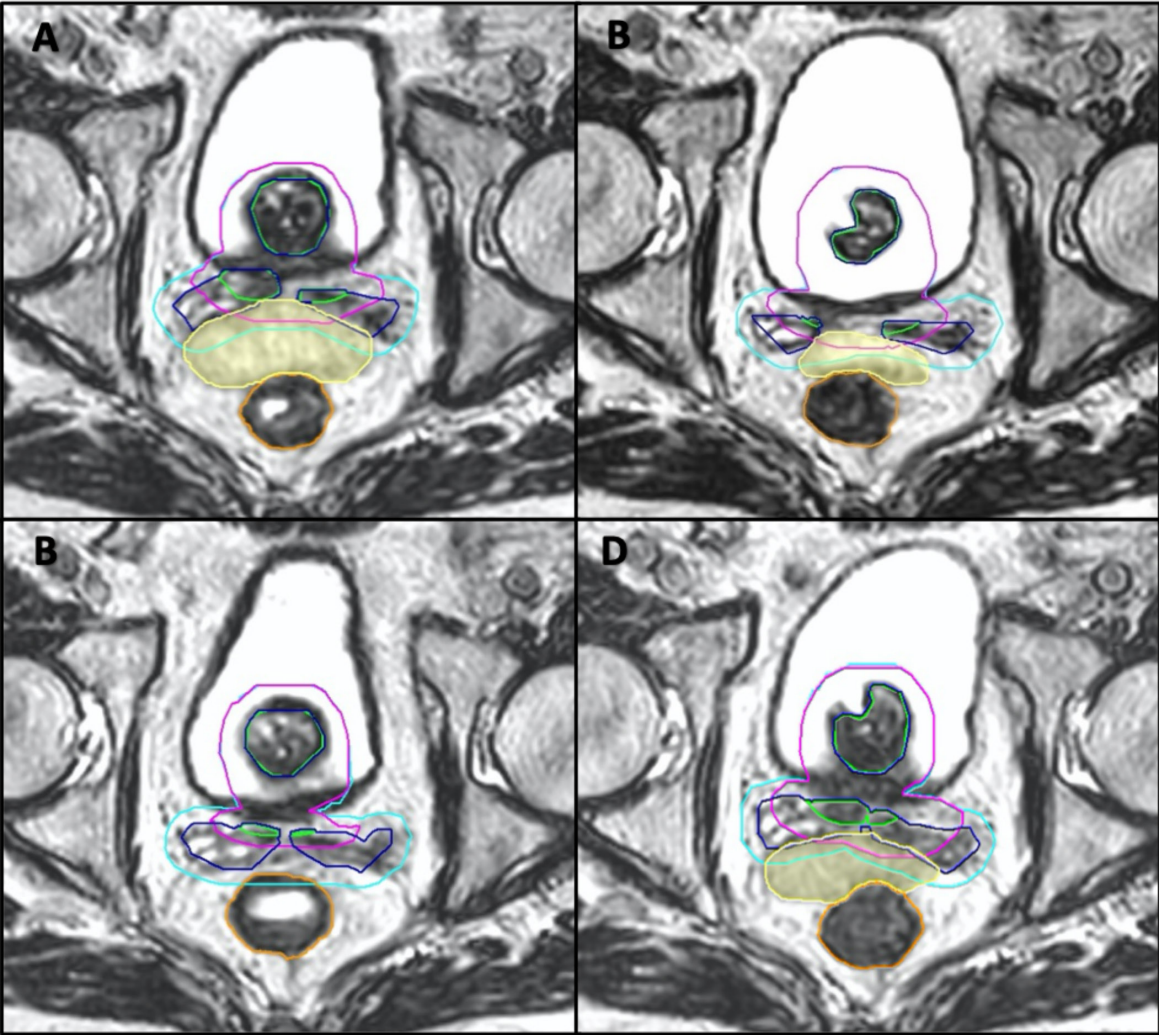
Small bowel movement during the course of treatment. These four images demonstrate four separate fractions of a 20-fraction prostate cancer radical treatment. These axial images, taken at the level of the mid-femoral heads, demonstrate interfractional motion of the organs at risk, particularly that of the small bowel which is shaded in yellow. Various target contours are denoted in purple and aqua (PTVs) and green/blue (prostate and SV); the rectum is outlined in orange. In three fractions (**A, C, D**), the small bowel is sitting adjacent to the prostate and seminal vesicles and thus overlapping in various degrees with the PTV whilst in fraction C it has moved away out of the treated volume.

Online ART modifies the treatment based on changes in the anatomy on the day of the treatment. This will account for interfractional movement, changes to the target organ and varying shape and size of the organs at risk during radiotherapy; this is demonstrated in Figure 2, where the small bowel is seen to be moving in and out of the radiotherapy field on different days. The target itself may shrink during a course of treatment (*e.g.* cervix cancer) or the target may deform^30^ or swell during hypofractionated treatments.^31^ With daily adaptive recontouring and replanning, the need for rigid immobilisation and invasive tracking methods such as fiducial markers become redundant. This can lead to a shortening of the patient workflow and improvement of the patient experience.

The potential benefits highlighted above of online ART may become more pertinent in hypofractionated treatments. Hypofractionation is likely to become standard of care for some tumour types and has been shown to be effective in curing some of the more common tumour sites; prostate,^6,32^ lung^33^ and breast.^34^ With fewer fractions, the precision of radiotherapy delivery with each fraction becomes more important. MRIgRT may have a role to play in this context; the HERMES trial^35^ is currently studying two fraction versus five fraction MRgRT for prostate cancer. With only two opportunities to deliver the intended dose, daily anatomical correction is considered mandatory. It is conceivable therefore that with ART, the overall dose to the target will increase and the dose to the organs at risk can be reduced, leading to a reduction in toxicity.

There is a limited capacity on MR linac machines, relative to standard machines and treatment times are longer. Therefore, informed by further research, patients and tumour types who will reap the most benefit from adaptive radiotherapy need to be prioritised.

#### Real-time imaging

Real-time cross-sectional imaging during treatment is a feature not commonly available with standard radiotherapy treatments. This allows monitoring of intrafractional movement of the target and organs at risk during treatment, thus giving the opportunity to gate treatment if necessary, such as pausing treatment when rectal gas passes through the rectum displacing the prostate.^36^ The prostate can move during treatment, either independently or due to increased bladder filling and/or movement of gas through the rectum. This movement has the potential to impact the dosimetric coverage. Studies have shown prostate displacement to occur during treatment; in one study a shift of >3 mm was seen over approximately 13% of the treatment time.^37^ A more recent study using data from patients treated on the MR-Linac for prostate cancer show only small anatomical displacements (<3 mm) in most patients. However, in those where larger displacements took place, the dose delivered was substantially different to that intended.^38^ It is anticipated that tracking and trailing of dose (*i.e.* dose follows the target if it moves) will soon be a reality for commercial MRIgRT systems.

Current IGRT techniques on standard linacs do not account for this movement and it is largely mitigated (in terms of target coverage) by the PTV margins. Cyberknife (Accuray, Sunnyvale), however, accounts for intrafraction movement by tracking fiducials with kV imaging throughout treatment. Yet, these treatments are sometimes lengthy and require the invasive procedure of inserting fiducials, and thus will not be suitable for all patients.

#### Radiation exposure

The use of MRI for image guidance removes the additional radiation exposure delivered from X-ray-based image guidance. MRIgRT thus allows frequent verification as well as continuous ‘real-time imaging’ radiation free. The dose from daily X-ray based image guidance (8–18 mGy daily) may be considered negligible in the setting of delivering large doses of curative radiation but continuous ‘real-time’ X-ray tracking daily over a course of treatment may increase this dose to a more clinically meaningful level.

The lack of additional radiation exposure with MRI may make this an ideal treatment modality for paediatric patients^10^ for whom secondary malignancy risk is a key concern. This benefit will need to be weighed against potential drawbacks such as anaesthetising a child, if needed to ensure tolerability, and technical considerations such as MR safe anaesthetic equipment.^39^

### Limitations

#### Technical considerations

MR imaging, although superior to X-ray-based imaging, is also susceptible to external factors affecting quality such as random motion.^40^ Ensuring a high geometric fidelity of the MR images is also paramount, impacting dose calculation and spatial accuracy of the target and organs at risk.^41,42^ Patients with some metal implants may be unsuitable due to distortions affecting the image quality or safety.

The electron return effect or Lorentz force is where secondary electrons move in a circular manner due to the existence of a magnetic field.^43^ This effect is especially evident around the point at which the beam exits and at tissue and air interfaces.^24^ This can impact the dose delivered,^44^ more so in certain tumour types such as whole breast^45^ and needs to be accounted for during the planning process.

MRIgRT systems currently do not allow non-coplanar beans or electron beams; both of which are used for multiple tumour types. Whilst dosimetric benefit of non-coplanar beams is not seen for all tumours, this does limit potential solutions for difficult plans.

#### Size limitations

The bore size of the MR-Linacs is fixed; the Elekta Unity MR Linac is 70 cm in diameter and closed. Patients who are larger in habitus, or those with significant claustrophobia, will not be suitable for treatment. The bore size can also limit the range of positions that can be reproduced.^26^

The maximum field size in the craniocaudal patient direction on the Elekta Unity MR-Linac is 22 cm which is too small for some patients needing lymph node irradiation in the pelvis^46^ or other longer fields. With this field size, 80% of plans would be suitable for the MR-Linac with a 1 cm margin; all prostate and brain patients were found to be suitable. However, this falls to 61% with larger tumour volumes such as cervix and some head and neck plans.^47^

#### Workflow and patient experience

Treatment times are much longer in duration compared to a standard linac; up to 1 hour has been reported for some sites and the average treatment duration for prostate cancer is 45 min^46^ in comparison to current treatment times on a standard linac with IMRT of approximately 10 min. Approximately 5% of patients have found the treatment on an MR linac lengthy.^48^ This is due to the time taken for recontouring by the clinician, optimisation of the plan to meet the constraints on the day and dose delivery.

The long treatment times may lead to greater intrafraction motion and thus the original plan created may no longer be valid and a positional shift and repeat optimisation of the plan may need to take place, further increasing the treatment time. Enhancing patient comfort on the couch is therefore crucial. Patients are provided with noise reducing headphones through which music can be played and interaction can occur between the patient and radiographers. Overall, the patient experience has been encouraging but the most frequently reported complaints were of noise (this was the most common complaint), parathesia and cold.^49,50^

Patients who complain of claustrophobia will be unsuitable for treatment on the MR linac as well as those who have contraindications to MR imaging such as cardiac implants.^51^

#### Resource intensive

Delivering each treatment on the MR-Linac requires a multidisciplinary team; a radiation oncologist, radiographers, and a physicist in comparison to treatment on a standard linac which usually only requires radiographers. For this reason, at The Royal Marsden Hospital, the standard MR Linac day would only treat up to seven patients a day, whereas on a standard linac the throughput is much higher. Work is underway to improve efficiency and reduce the number of staff needed at the console, *e.g.* training radiographers to contour^52^ and to plan. The use of this new technology will also require additional training for all staff in areas such as MR safety and MR anatomy.

#### Cost

The adoption of MR-Linacs is increasing rapidly but machines remain limited in number. The cost of MRIgRT systems is much greater than conventional linacs and this is due to a combination of the initial capital cost but also the cost of preparation of the site and service contracts. Radiofrequency shielding is an additional cost, which is not required for conventional linacs.

## MR-Linac in clinical practice at our institution

At our institution, an Elekta (Elekta AB, Stockholm, Sweden) Unity MR-Linac has been in clinical use since 2018. Patients are only treated within a clinical trial. We recruit all patients to the MOMENTUM study,^53^ which is a collaborative international database collecting technical, imaging and patient data on over 2000 patients to date. Initially, in prostate cancer, we also conducted the PRISM study,^54,55^ treating a total of 27 patients with intermediate risk prostate cancer with 20 fraction treatment. Currently, prostate patients treated on the MR-Linac mostly receive five fractions.

**Figure 3.**
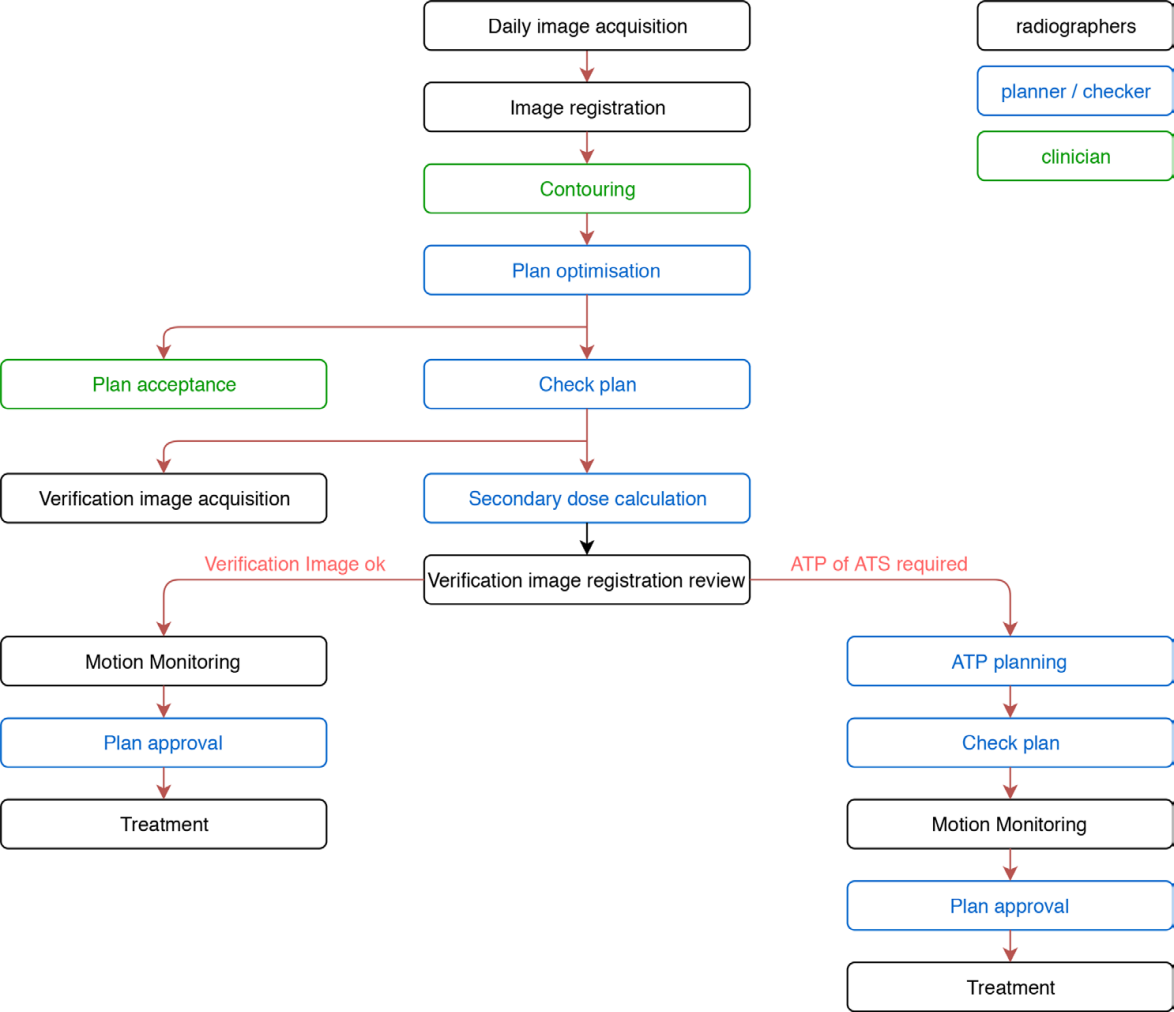
Schematic of a treatment workflow on the MR Linac at the Royal Marsden Hospital (Figure adapted from prototype by Alex Dunlop and Helen McNair, RMH).

**Figure 4.**
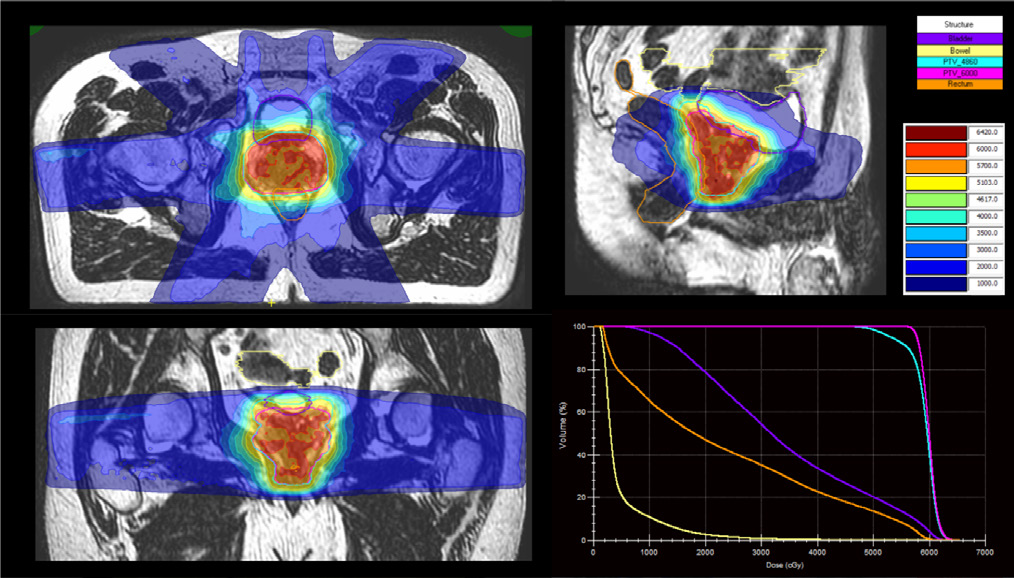
An example of a daily plan on the MR linac for a patient with prostate cancer receiving 60 Gy in 20 fractions. Sagittal, coronal, axial images and a dose–volume histogram are demonstrated; 57 Gy colourwash denoted in orange.

The workflow for treatment is shown in Figure 3. The online adaptive workflow produces a new radiotherapy plan for patients, based on their anatomy, for every fraction (Figure 4). Once the patient is set up in the correct position, a session image is acquired. The clinician or trained radiographer reviews the image and assesses for a change in anatomy. If present, the target, and/or organs at risk contours are modified or recontoured and reoptimisation of the plan is carried out by the physicist. A verification image is obtained to assess for intrafractional movement. If significant, a positional shift is applied to the new plan (‘Adapt-to-Position’) to ensure target coverage. The workflow for the Viewray MRIdian differs slightly and more details can be found in Tocco et al.^46^

## Future

### MR-only workflow

MR-only workflow describes the procedures needed for radiotherapy planning based solely on MR, without a CT planning scan. This may reduce hospital visits for the patient by negating the need for pre-treatment planning imaging. It will also reduce costs and lower the total patient radiation dose.^41^ It will also overcome any contouring discrepancies caused by the introduction of systematic errors from inaccurate co-registration of the planning CT and MRI during the pre-planning process.^46,56^ In prostate cancer, this arises usually from a discrepancy in bladder and rectal volumes between the two scans.

The main necessary step in implementation is the generation of synthetic CTs to provide electron density information to enable dose calculation^26^ which require high geometric accuracy of the MR images. MR-only workflows have been shown to be feasible and have similar dosimetric accuracy as CT-based electron density planning in pelvic cancers.^57^ Clinical implementation of this approach is in process.

## Autosegmentation

Currently, contours are automatically propagated from the initial MR to the session MR on the day of treatment following deformable registration. Most radiation oncologists then modify these contours rather than starting from scratch. Autosegmentation may improve the workflow in the future by reducing the time needed for delineation and even avoiding the need for recontouring. This will also accelerate time to beam-on, reducing time for motion to occur.

### Expansion of clinical roles

The role of the radiographer is likely to expand to take the lead in the online workflow. This may reduce the need for a clinician to be present during treatment and could improve patient throughput. A ‘clinician-lite’ approach has been adopted at the Christie for simple prostate treatments^58^ and at our institution, we are in the process of training the radiographers to perform online contouring. Pathmanathan et al demonstrated good agreement between radiographer contours and the gold-standard on MRI.^21^

### Real-time imaging

Real-time imaging during radiotherapy can be invaluable for some tumour types. It offers the possibility of adjusting the patient, or even pausing treatment, when the internal anatomy changes during treatment leading to the target moving out of the field or when an organ at risk moves into a high dose area; this can occur in cases of peristalsis, air in the rectum and breathing.

Logically, one would presume that if there is a benefit for MR-guided adaptive radiotherapy, it would be largest for ultra-hypofractionation, due to the inclusion of MR guidance and in-beam imaging. Level I evidence is however currently lacking to demonstrate its benefit.

The ultimate goal would be that of intrafraction adaptive replanning whereby a plan is being adapted during beam delivery,^59^ and thus potentially negating the need for a PTV margin. This would be especially useful in ultra-hypofractionated regimens^60^ and would be expected to reduce toxicity of treatment.

### Dose escalation and reirradiation

In prostate cancer, the dominant intraprostatic lesion (DIL) is known to be the most common site of local relapse. These can be visualised on MR sequences which offers the possibility of dose escalation under direct vision, expecting that this will lead to greater tumour control.^61,62^ MRIgRT with adaptation offers the opportunity of reirradiation of tumours with reduced margins.

### MR imaging during treatment

The MR-Linac offers the ability to collect multiple MRI images daily, before and during treatment. The quality of these images are not as high as those obtained from a 3T diagnostic MRI machine, yet it provides a wealth of data which otherwise would have been difficult to obtain, as daily diagnostic MR imaging is expensive and time consuming. This information will enable radiation oncologists to study changes in tumours during the course of radiation treatment and carry out dose–response studies.

One of the more exciting possibilities of MR-guided radiotherapy is the ability to perform functional imaging such as diffusion-weighted imaging (DWI) during treatment.^63^ DWI is sensitive to the Brownian motion of water within tissues and can be used to discriminate malignant from benign tissue. Malignant tumours have a low ADC value.^64^ DWI images are also used to monitor response to treatment, post-chemotherapy or radiotherapy, to differentiate post-therapy changes from active tumour and to detect recurrent tumour.^65^

This can provide information about the biology about the tumour and may act as a predictive biomarker for certain tumour sites as well as an indicator of tumour response to treatment.^66^ It has been demonstrated that changes on DWI images may be useful for prediction and early assessment of pathologic response to radiotherapy with a better accuracy than volumetric measurements in rectal cancer.^67^ In prostate cancer, the ADC values have been shown to increase in the initial few weeks of therapy, more markedly in those patients who have better clinical outcomes.^68–70^ The same pattern has been seen in other tumours sites.^71,72^ ADC as an imaging biomarker is sensitive but lacks specificity; it can be affected by factors such as necrosis and altered vasculature.^73^

Obtaining functional MR images could be done during the online workflow, hence would not take additional time. With these images, daily response assessment can be carried out which can influence the dose delivered to parts of the organ; *e.g.* dose escalation to areas with persistent restricted diffusion^24,46^ as these areas are likely to harbour a more radioresistant phenotype of the tumour. The MR-Linac has been shown to identify these areas in some tumours.^74^ The clinical value of these biological markers are yet to be determined and needs further exploration.

## Conclusions

MR-guided radiotherapy remains in its early stages but is exciting and rapidly evolving. It offers greater possibilities for image-guided radiotherapy, thus offering opportunities for dose-escalation and reduction in toxicity. The development of individualised treatment plans is possible due to the combination of superior soft tissue contrast, real-time imaging and online daily adaptation. We may see a reduction of margins, increase in the dose per fraction and the use of functional data to guide treatment. However, at present, it remains resource and time intensive to deliver and is not yet widely available. Although the theoretical possibilities of this new technology is numerous, prospective randomised clinical trials and extensive clinical validation are required before clear benefits for MRIgRT can be claimed.
